# Hydroxychloroquine on the Pulmonary Vascular Diseases in Interstitial Lung Disease: Immunologic Effects, and Virus Interplay

**DOI:** 10.3390/biomedicines10061290

**Published:** 2022-05-31

**Authors:** Jun-Jun Yeh, Shih-Hueh Syue, Yi-Fun Sun, Yi-Ting Yeh, Ya-Chi Zheng, Cheng-Li Lin, Chung Y. Hsu, Chia-Hung Kao

**Affiliations:** 1 Department of Family Medicine, Geriatric Medicine, Chest Medicine and Medical Research, Ditmanson Medical Foundation Chia-Yi Christian Hospital, Chiayi 600566, Taiwan; anvin.funlan@msa.hinet.net; 2 College of Medicine, China Medical University, Taichung 406040, Taiwan; orangechengli@gmail.com (C.-L.L.); hsucy63141@gmail.com (C.Y.H.); 3 Department of Family Medicine, Ditmanson Medical Foundation Chia-Yi Christian Hospital, Chiayi 600566, Taiwan; snowberry2626@gmail.com (S.-H.S.); 07712@cych.org.tw (Y.-F.S.); st8111110@gmail.com (Y.-T.Y.); cych07440@gmail.com (Y.-C.Z.); 4 Management Office for Health Data, China Medical University Hospital, China Medical University, Taichung 406040, Taiwan; 5 Graduate Institute of Biomedical Sciences, College of Medicine, China Medical University, No. 2, Yuh-Der Road, Taichung 406040, Taiwan; 6 Department of Nuclear Medicine and PET Center, China Medical University Hospital, China Medical University, Taichung 406040, Taiwan; 7 Department of Bioinformatics and Medical Engineering, Asia University, Taichung 41354, Taiwan; 8 Center of Augmented Intelligence in Healthcare, China Medical University Hospital, China Medical University, Taichung 406040, Taiwan

**Keywords:** hydroxychloroquine (HCQ), pulmonary vascular disease (PVD), interstitial lung disease (ILD)

## Abstract

To investigate the effects of hydroxychloroquine (HCQ) drug use on the risk of pulmonary vascular disease (PVD) in an interstitial lung disease cohort (ILD cohort, ILD+ virus infection), we retrospectively enrolled the ILD cohort with HCQ (HCQ users, N = 4703) and the ILD cohort without HCQ (non-HCQ users, N = 4703) by time-dependence after propensity score matching. Cox models were used to analyze the risk of PVD. We calculated the adjusted hazard ratios (aHRs) and their 95% confidence intervals (CIs) for PVD after adjusting for sex, age, comorbidities, index date and immunosuppressants, such as steroids, etc. Compared with the HCQ nonusers, in HCQ users, the aHRs (95% CIs) for PVD were (2.24 (1.42, 3.54)), and the women’s aHRs for PVD were (2.54, (1.49, 4.35)). The aHRs based on the days of HCQ use for PVD of 28–30 days, 31–120 days, and >120 days were (1.27 (0.81, 1.99)), (3.00 (1.81, 4.87)) and (3.83 (2.46, 5.97)), respectively. The medium or long-term use of HCQ or young women receiving HCQ were associated with a higher aHR for PVD in the ILD cohort. These findings indicated interplay of the primary immunologic effect of ILD, comorbidities, women, age and virus in the HCQ users.

## 1. Introduction

**Figure 1:**
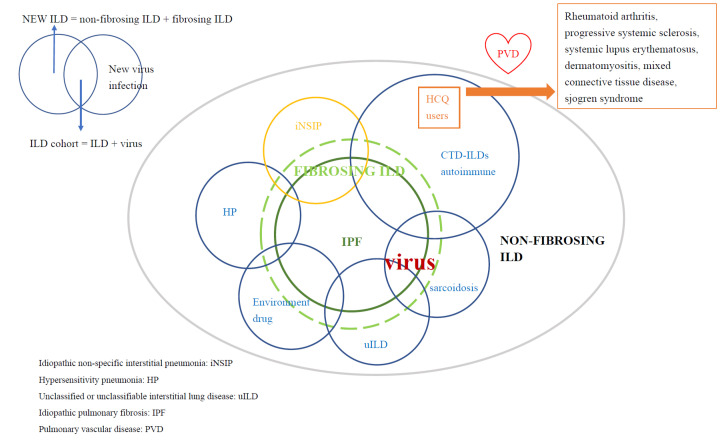
Illustration of interstitial lung disease and virus infection (ILD cohort).

Progressive fibrosing interstitial lung disease (PF-ILD) is another form of interstitial lung disease (ILD) and consists of a diverse group of ILDs characterized by a different amount of inflammation and fibrosis, frequent disease exacerbation and earlier mortality [1,2]. ILD, especially PF-ILD, may develop into pulmonary heart disease, including pulmonary embolism, pulmonary artery hypertension (PAH), and pulmonary circulation disease—pulmonary vascular diseases (PVD) [3,4,5]. Meanwhile, infections, such as those that are viral, may be concurrent with or associated with the initiation or exacerbation of ILD; this vicious coexistence may also contribute to PVD (Figure 1) [3,5,6,7,8,9].

The PAH is characterized by excessive proliferation and apoptosis resistance in pulmonary artery smooth muscle cells. Loss-of-function mutations in the bone morphogenetic protein receptor type-II (BMPR2) are the most common cause of heritable PAH. Hydroxychloroquine (HCQ) both inhibited the autophagy pathways and increased the expression of BMPR2 via lysosomal inhibition. The combination of these factors and the administration of HCQ prevented the development of PAH, and vascular remodeling after monocrotaline, and prevented the progression of established PAH in an experimental study [10]. However, the prolonged use of HCQ has been implicated in the development of conduction disturbances and myocardial dysfunction, leading to heart failure [11]. Owing to the cross-reaction of the heart and lung, heart failure was predisposed to PAH [6]. Moreover, long-term HCQ use may contribute to respiratory muscle weakness, leading to RF [12]. The adverse reaction of HCQ is displayed in Table A1 in Appendix A.

**Figure 2:**
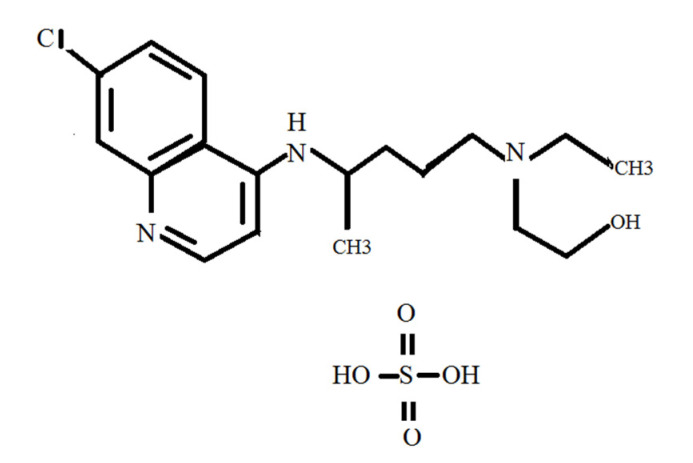
Hydroxychloroquine chemical structure.

Taken together, HCQ plays a contrasting role for PVD in ILD, especially with long-term use. The HCQ chemical structure display in Figure 2.

In the virus era, the virus may play a critical role in ILD [13]. Furthermore, HCQ may be associated with a lower incidence of bacterial infection in ILD [14,15]. However, the relationship between HCQ and viral infections is controversial [16,17]. Up to date, no studies have investigated HCQ use on the risk of PVD among ILD patients with viral infections. Therefore, in the present study, we examined the effects of HCQ drug use on the risk of PVD in the ILD cohort (ILD + virus infection) in the general population.

## 2. Material and Methods

### 2.1. Patient and Public Involvement

We utilized data from the National Health Insurance Research Database (NHIRD) of Taiwan. It was established when a single-payer compulsory social insurance system was launched in Taiwan in 1995, which contains the medical records of almost 99% of 23 million Taiwanese residents. The Longitudinal Health Insurance Database, which consists of files on ambulatory claims, inpatient claims and prescriptions for 1 million insured individuals, was used as a data source in the present study. The disease codes follow the *International Classification of Disease, Ninth Revision, Clinical Modification* (ICD-9-CM).

#### 2.1.1. Identifying the ILD Cohort

**Figure 3:**
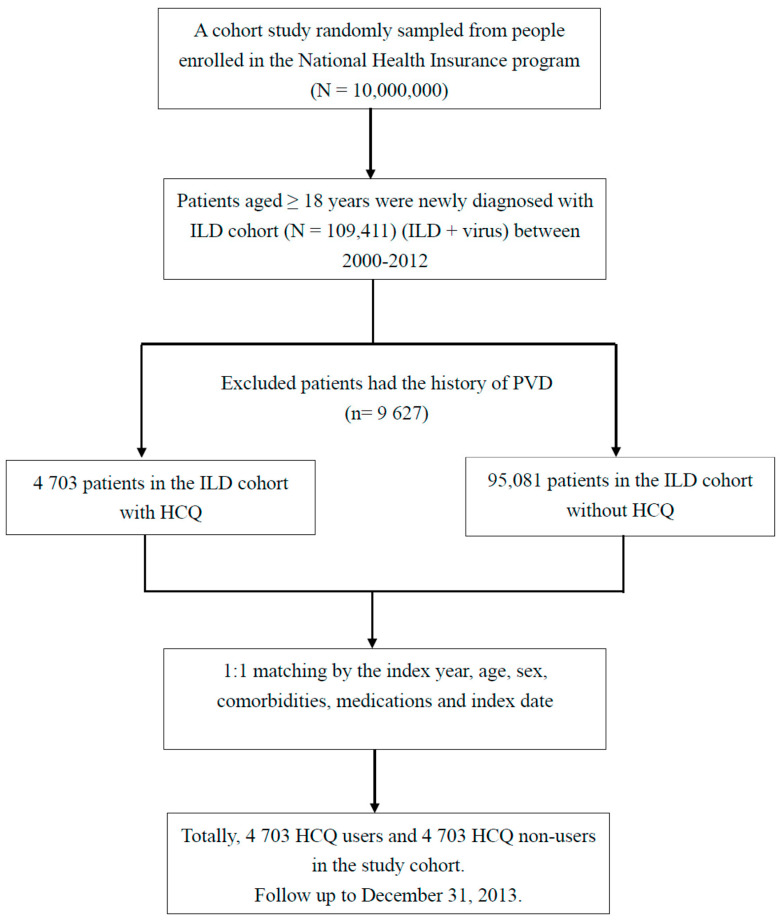
Flow chart of the selection of the ILD cohort.

Initially, we identified ILD patients (new ILD) and virus infections (new virus infection). For this cohort study, patients with two or more outpatient visits or one hospitalization for ILD (ICD-9-CM code: 135, 237.7, 272.2, 277.3, 277.8, 500–505, 506.4, 508.1, 508.8, 515–516, 446.21, 446.4, 495, 517.2, 517.8, 518.3, 555, 710, 714.81, 720, 759.5) in the period of 2000 to 2012 were entered into the study. The new virus infection included ICD-9CM: 0.42, 0.53, 070.20, 070.22, 070.30, 070.32, 070.41, 070.44, 070.51, 070.54, 0.75, 078.5, 079.0–079.5, 079.81–079.83, 079.88–079.89, 480, 486–488 [7,8,9,18]. Patients aged ≥18 years who had a new ILD, or new virus infection were selected for the ILD cohort. The ILD cohort includes the ILD + virus infection. The index date of a new virus infection is the date of the ILD cohort. Patients with an age <18 years or a history of PVD before entry into the study were excluded (The full names of the ICD-9-CM codes are listed in Table A1 and Table A2 in Appendix A) (Figure 1 and Figure 3) [9,19,20,21].

##### Diagnosis Progressive Fibrosing-ILD

Idiopathic pulmonary fibrosis (IPF) is a prototype of PF-ILD with a poor prognosis. However, PF-ILD other than IPF, such as idiopathic nonspecific interstitial pneumonia (iNSIP), fibrotic hypersensitivity pneumonitis (HP), connective tissue diseases (CTD)-ILD or autoimmune-ILD, also have a progressive phenotype, manifesting as a worsening of dyspnea, decline in lung function, and increased extent of fibrosis on high-resolution computed tomography (HRCT), with high morbidities, such as PVD. In Taiwan, we defined PF-ILD as developing any of the following within 2 years of diagnosis: (1) a relative decline in forced vital capacity (FVC) ≥ 10%; (2) a relative decline in diffusion capacity of carbon monoxide (DLCO) ≥ 15%; (3) worsening of radiological appearance or symptoms and a relative decline in FVC from 5~10% [1,2].

Similar to our study, the Yang et al. study found that IPF patients have concurrent pneumonia, such as cytomegalovirus infection, influenza and PVD, which were the critical cause for admission based on the NHIRD [8]. These studies support our speculations (Figure 1 and Figure 3).

#### 2.1.2. Application of HCQ Use

The application of HCQ includes autoimmune diseases or CTD, such as lupus. The indication of HCQ is for patients with autoimmune or CTD-lupus, not viral infections. For example, HCQ had a positive impact on lupus on robust outcomes, such as accrual damage, disease activity and survival, and also pleiomorphic effects, including a decrease in the need for steroids, attenuation of neonatal lupus, increased insulin sensitivity, lower fasting glucose and protection against thrombotic risk, dyslipidemia, and perhaps infections, etc.

The CTD-ILD or autoimmune-ILD in the HCQ user includes rheumatoid arthritis, systemic lupus erythematosus, Sjogren’s syndrome, scleroderma, dermatomyositis and mixed connective tissue disease.

**Figure 4:**
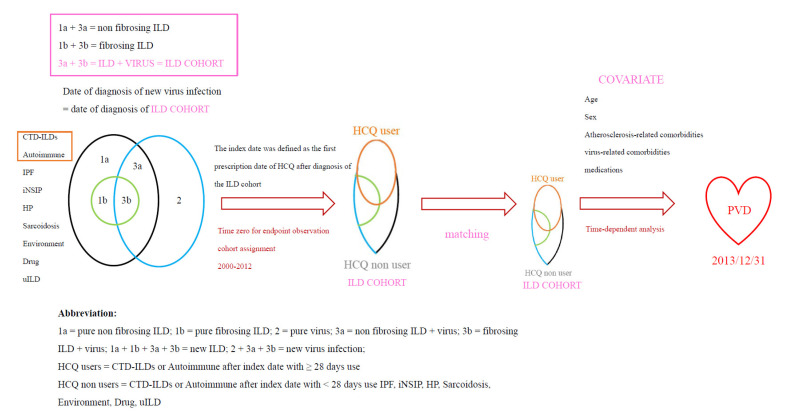
Selection of the patients for Hydroxychloroquine users among the interstitial lung disease with virus infection (ILD cohort).

However, CTD-lupus, autoimmune disease, environmental lung disease, and their -virus-related comorbidities diseases, such as cancer and virus infection may coexist. Thus, we clustered these groups of patients (ILD + virus infection) into the ILD cohort (Figure 1 and Figure 4).

#### 2.1.3. Definition of HCQ Users

The index date was defined as the first prescription date of HCQ after diagnosis of the ILD cohort. Slow onset of anti-inflammatory therapeutic efficacy is typical for HCQ as it may take up to 4 to 6 weeks for the onset, and 3 to 6 months to achieve maximal clinical efficacy [11]. Thus, we defined the case cohorts (HCQ users) as patients who used HCQ therapy for ≥28 days after the index date. Patients who never underwent HCQ therapy or drug use of <28 days were the controls (HCQ nonusers) in the present study and they were randomly assigned index dates between 2000 and 2012. In one case, the patient was then matched to one control patient through propensity score matching. The propensity scores were calculated using a logistic regression model, which included age, gender, comorbidities, medication and index date. The end of the study was 31 December 2013. Data for patients who withdrew from the insurance program or who died were omitted from the analyses (Figure 1 and Figure 4).

### 2.2. Data Availability Statement

The Taiwan Ministry of Health and Welfare (MOHW) holds the dataset in this study. The MOHW must approve our application to access the current data. Any researcher who is interested in accessing this dataset can submit an application form to the MOHW requesting access (MOHW, Email: stcarolwu@mohw.gov.tw). The Taiwan Ministry of Health and Welfare Address is No.488, Sec. 6, Zhongxiao E. Rd., Nangang Dist., Taipei City 115, Taiwan (R.O.C.). Phone: +886-2-8590-6848. All relevant data are within the paper.

### 2.3. Ethics Statement

The NHIRD encrypts patients’ personal information for protecting privacy. Meanwhile, the NHIRD provides researchers with anonymous identification numbers which are associated with relevant claims information, such as sex, date of birth, prescription of medications and the reception of medical services. Therefore, when the researcher accesses the NHIRD, they do not need patient consent. This study was approved to fulfill the condition for exemption by the Institutional Review Board (IRB) of China Medical University (CMUH104-REC2-115-CR6). Moreover, the IRB specifically waived the consent requirement.

### 2.4. Main Outcome and Comorbidities

The primary outcomes were newly diagnosed PVD (ICD-9-CM code 415–417) within 1 year of the new viral infection [8,18]. These virus infections and atherosclerosis may play a critical role in PVD in the ILD cohort. Therefore, atherosclerosis-related comorbidities, such as diabetes and retinopathy, hypertension, hyperlipidemia, alcohol-related disease, chronic kidney disease, coronary artery disease, stroke and gout, and virus-related comorbidities, such as chronic obstructive pulmonary disease (COPD), sleep disorder, cancer, and mental disorder were entered into this study. We also included medications, such as colchicine, inhaled corticosteroids (ICSs), oral steroids (OSs), aspirins, statins, warfarin, clopidogrel, nonsteroidal anti-inflammatory drugs (NSAIDs) and immunosuppressants, such as cyclophosphamide (CYC), azathioprine (AZA), and methotrexate (MTX) for inflammation in the ILD cohort in adjustments. The full names of the comorbidities and medications are listed in the Appendix table.

### 2.5. Diagnosis of Pulmonary Artery Hypertension

In Taiwan, the criteria of the PAH included: (1) a resting elevated mean pulmonary artery pressure (m PAP) of ≥25 mmHg, measured during right heart catheterization; (2) the end-expiratory pulmonary artery wedge pressure (PAWP) of ≤15 mm Hg with a pulmonary vascular resistance (PVR) of >3 Wood Units. In this study, the criteria of PVD include: (1) having: ≥2 claims for PVD [ICD-9-CM: 415–417]; (2) after the initial PVD claim (index date) the patients need to have ≥1 claim for pulmonary embolism ≤12 months prior or 1 month after. Both cohorts were required to have: (1) prior to any PVD claim, the patients need to have ≥1 claim for right heart catheterization within 6 months, or (2) prior to a specialist-diagnosed PVD claim, the patients need to have ≥1 claim for an echocardiogram within 6 months [22].

### 2.6. The Primary Effect of CTD-ILD in Relation to PVD

The PVD may be found in any stage of the course of CTD-ILD or autoimmune disease-related ILDs with or without fibrosing [23,24]. Meanwhile, the PVD, in the late course of lupus with PF-ILD, was life-threatening [25,26]. In the recent Chiu et al. study, they found that (20/150, 22%) with HCQ use and (17/150, 11.3%) with PAH in the 150 patients had ILD (33/150, 33% fibrotic predominant, PF-ILD) [2]. In this study, fibrosing CTD-ILD or IPF was grouped as fibrosing-ILD. However, the IPF was excluded from the HCQ users.

### 2.7. The Combination Effects of Comorbidities, Virus Infection and CTD-ILD in Relation to PVD

The PVD in the CTD-ILD may be primary or secondary to chronic obstructive pulmonary disease (COPD), tumor or medications [27]. Meanwhile, PVD may present as acute (ICD-9 CM 415) or chronic (ICD-9 CM 416). These comorbidities, such as COPD, CAD (left heart failure) and cancer were entered into the analysis in this study. Virus infection, such as human immunodeficiency virus (HIV), could play a role in the PVD formation and may present as acute PVD or perhaps chronic PVD [28,29]. The duration between the virus infection and symptoms of PVD may be short or long, such as from 6 months to 1 year [24,30,31]. Meanwhile, hepatitis with interferon treatment may develop into chronic PVD [29,32]. Similar to that, the long-term effects of the herpes virus may be chronic PVD in ILD [29,33]. In a recent ongoing study, the long-term effects of coronavirus (long-COVID) are associated with chronic PAH and seem to be in accordance with our postulations [34]. Thus, the newly diagnosed PVD (ICD-9-CM code 415–417) within 1 year of new viral infection was entered into the study.

### 2.8. HCQ Concentration

Owing to the laboratory data being unavailable in NHIRD, subjects who discontinued HCQ (particularly those with low education) may have been non-adherent with other medications (e.g., prednisolone) and physician advice, perhaps due to mistrust or not understanding physician recommendations. Meanwhile, HCQ toxicity is related to the duration of use; steroids enhance HCQ potency, and the HCQ accumulated in the neutrophil. These findings indicated HCQ could play a critical role in the management of the chronic PVD [35,36,37]. Therefore, immunosuppressants, such as OSs, and the duration of HCQ use and frequency of discontinuation could replace the severity of the ILD and HCG concentration for the management of the chronic PVD.

### 2.9. The Downbeat Role of Virus in ILD (ILD-Virus Coexist)

Based on (1) the meta-analysis study, the pool prevalence of the virus infection in IPF was high, up to 53.72% [7]. (2) Viral infections, such as human immunodeficiency virus, Epstein-Barr virus, rhinovirus, influenza and coronavirus are associated with ILD and PVD, and the bidirectional reaction of viral infections and CTD/autoimmune disease were found [9,19,38,39,40]. (3) IPF and CTD-ILD (especially, PF-ILD) shared nearly the same comorbidities, such as cancer, heart disease, PVD and virus infection. (4) These diseases may share similar cellular pathology [1]. (5) Virus infections and acute exacerbation of CTD-ILD can mimic one another, can coexist, and can promote each other—a ‘vicious’ coexistence. It is reasonable, then, that we included them in the ILD cohort in this study. Therefore, in this ILD cohort, we included the (ILD + virus) (Table A2 in Appendix A and Figure 1, Figure 3 and Figure 4).

### 2.10. Statistical Analysis

The Chi-squared test and the Mann–Whitney test were used to compare categorical and continuous variables between the HCQs users and HCQs nonusers cohorts. We calculated the incidence rate based on 1000 person-years. For minimizing the selection bias due to non-randomized allocation during the study, we performed propensity score matching to balance the baseline characteristics, including age, sex, comorbidities, medications and year of an index between these two cohorts. If the subjects of the samples from the candidates have equally close propensity scores to the propensity score of the multiple subjects, one of those is randomly selected. The nearest-neighbour matching was performed, namely pre-specified calipers, and we used 0.2 of the standard deviation of the logit of the propensity score without replacement with a 1:1 matching ratio.

We matched each patient in the HCQ users cohort with a patient in the HCQs nonusers cohort by means of propensity scores. Meanwhile, we performed the time-dependent Cox models for comparing the risk of PVD in the propensity-matched HCQ users cohort (n = 4703) and nonusers cohort (n = 4703). Moreover, we used the Kaplan–Meier method to obtain the PVD incidence cumulative curves of these two cohorts, and these curves were subsequently examined by performing log-rank tests. The Cox proportional hazards model with time-dependent covariates was used for examining the adjusted hazard ratio (aHR) and 95% confidence interval (95% CI). A two-tailed *p*-value of <0.05 indicated statistical significance. All analyses were performed using SAS, version 9.4 (SAS Institute, Inc., Cary, NC, USA).

## 3. Results

We finally defined 4703 ILD patients as HCQ users in the present study. Table 1 lists the baseline characteristics of the HCQ users and nonusers. After matching was conducted, it was revealed that patients in the two cohorts were aged mainly below 49 years (~48%) and were predominantly women (~79%). With regard to the distribution of comorbidities, the HCQ cohort had more patients with cancer than the non-HCQ cohort. The proportions of patients using colchicine, ICSs, OSs, aspirin, statins, warfarin, clopidogrel, NSAIDs, CYC, AZA and MTX between the case cohort and the control cohort were similar.

The aHR for PVD in the HCQ users compared when compared to the non-HCQ patients was 2.24 (95% CI = 1.42, 3.54), which was statistically significant after age, sex, comorbidities and medications were controlled (see Table 2).

**Figure 5:**
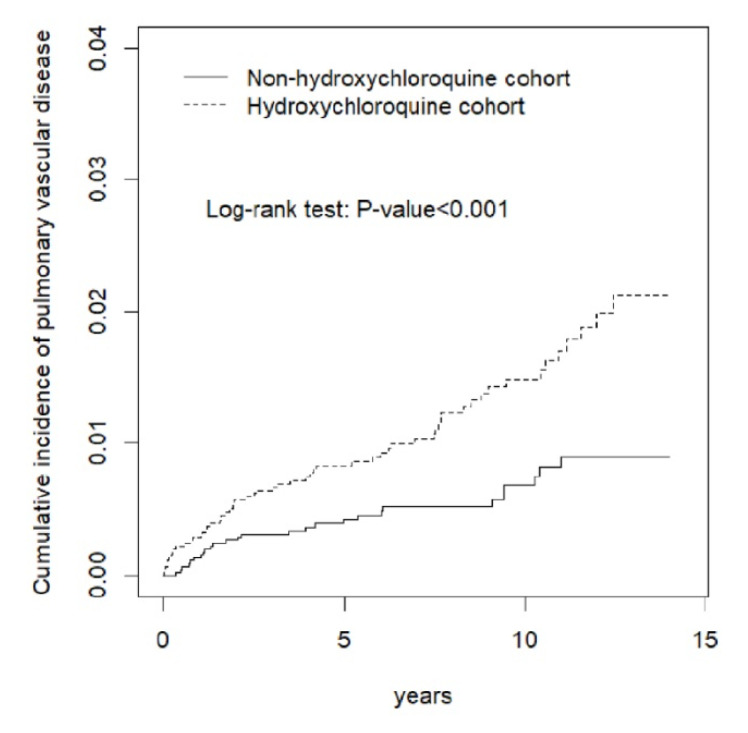
Cumulative incidence of pulmonary vascular disease curves for hydroxychloroquine users and hydroxychloroquine nonusers by propensity score matching.

The incidence rate of PVD in the HCQ cohort was 1.63 per 1000 person-years, which was also higher than the incidence rate in the non-HCQ cohort. A higher cumulative incidence curve of PVD was obtained from the HCQ cohort relative to the non-HCQ cohort, as illustrated in Figure 5.

Table 3 shows the association between HCQ and PVD in different age groups and sex. In the propensity score-matched cohort, the aHR of PVD was higher in HCQ users aged <49 years relative to HCQ nonusers. For women, in the propensity score-matched cohort, the aHR of PVD for the HCQ cohort compared with the non-HCQ cohort was 2.54 (95% CI = 1.49, 4.35).

The effect of the duration of HCQ therapy is shown in Table 4. Patients who used HCQ for more than 30 days had a higher aHR of PVD than the other users (31–120 days of HCQ use: aHR = 3.00, 95% CI = 1.89, 4.81; >120 days of HCQ use: aHR = 3.83, 95% CI = 2.46, 5.97). There was no association with PVD for 28–30 days use (aHR = 1.27, 95% CI = 0.81, 1.99).

**Figure 6:**
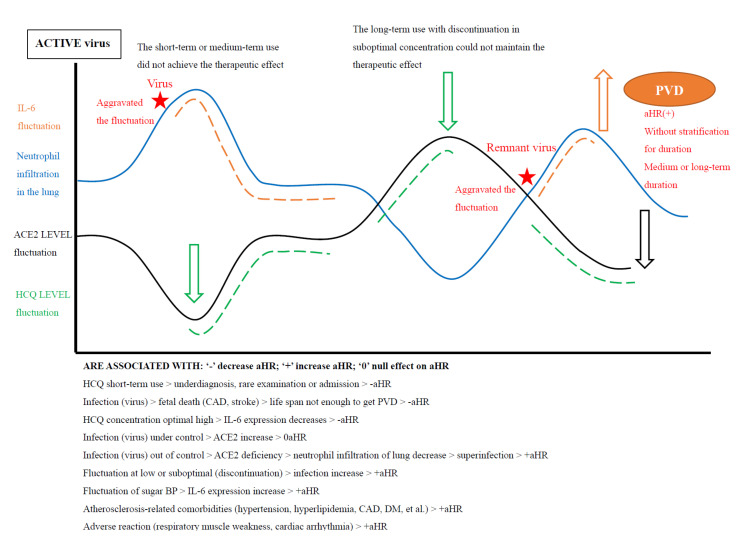
Dual effect of hydroxychloroquine on pulmonary vascular diseases. Illustration of the effect of fluctuation with hydroxychloroquine concentration on the risk of pulmonary vascular diseases. Illustration of the HCQ effect on the ILD with virus infection for pulmonary vascular diseases.

Owing to that the virus could be concurrent with or initiate or exacerbate the severity of ILD, we classified the scenario of virus infection in the discussion (Figure 6).

Figure 6 illustrate that if the virus could not be eradicated, chronic PVD may develop in the ILD cohort.

We display the speculatios and findings of this study in Table 5.

## 4. Discussion

### 4.1. Key Points of This Study

This is the first study in English literature to investigate the effect of HCQ on the risk of PVD among ILD with virus infection. The major finding of the present study is that HCQ is associated with a higher incidence of PVD. Second, the short-term use of HCQ has a null effect on aHR for PVD, the medium-term or long-term use with higher aHR for PVD. Third, the young woman with a higher aHR of PVD (Table 3).

### 4.2. HCQ Discontinuation for Higher aHR

A high frequency of HCQ discontinuation in CTD-ILD was found in the previous system review—the percentage of non-adherent patients ranged from 43% to 75% in lupus [41]. In a Korean study, Lee et al. reported that 48.9% (115/235) of lupus patients experienced HCQ discontinuation with poor adherence [42]. It is to be noted, however, that patients at the therapeutic target (stable optimal concentration of HCQ) throughout follow-up tended to have fewer flare-ups and had a lower incidence of lupus complications, such as vacuities, pericarditis, which were related to PVD [43,44]. Maximizing the dose of a currently prescribed HCQ in a low concentration may contribute to the fluctuation of the HCQ concentration and angiotensin-converting enzyme 2(ACE2)/ACE imbalance at a suboptimal level. Thus, this augmentation strategy could not prevent the flare-up and attenuate the incidence of complications [44,45]. Altogether, short-term or medium-term use did not achieve the therapeutic effect, and long-term use with discontinuation in a suboptimal concentration could not maintain the therapeutic effect. These findings may explain that HCQ use was associated with a higher aHR for PVD in the ILD cohort (See Figure 6, Table 5) [46,47]. A study by Brasil et al. concluded that maintaining HCQ was associated with a lower flare-up risk [47]. In contrast, even among lupus patients in remission, lowering or stopping HCQ was associated with a 2-fold increase in flare-up risk compared to HCQ maintenance [47]. These previous reports are in line with our speculations.

### 4.3. Comorbidities for Higher aHR

Moreover, atherosclerosis-related comorbidities, such as hypertension, hyperlipidemia and diabetes or virus-related comorbidities, such as cancer, diabetes, sleep disorder and mental disorder were predisposing factors to PVD [20]. For example, if hypertension or diabetes is poorly controlled, fluctuations of sugar or blood pressure may contribute to overexpression of interleukin-6 (IL-6)which were associated with oxidative stress-lung injury and PAH [48]. Thus, in medium-term or long-term use, the primary ILD effect and their comorbidities effects may overwhelm the protection effect of HCQ, leading to a higher aHR for PVD (See Figure 6, Table 5).

### 4.4. Short Life Span and Rare Detection for Lower aHR

As mentioned before, the achievement of the therapeutic concentration of HCQ for PVD may need 4–6 weeks or even >90~120 days, depending on the patient’s status [11]. As such, short-term use of HCQ may be without effect on aHR for PVD [49]. In contrast, the primary effect of ILD, comorbidities, may contribute to a higher aHR of PVD. However, there are two factors that contribute to lower aHR, including: (1) PVD may present as coronary artery disease (32.7%) with heart failure and expire; owing to these diseases, these patients did not have an adequate life span for PVD in the short duration use. (2) The short-term use of patients with rare admission for acute exacerbation with a paralleled rare frequency of detection of PVD. These confounding factors may lead to a lower frequency of diagnosis of PVD [50]. These combined factors may explain how the short-term HCQ use was with a null effect on aHR for PVD in the ILD cohort.

### 4.5. Virus Infection for Higher aHR

Notably, persistent or pre-existing ACE2 deficiency with a high level of IL-6 enhanced neutrophil infiltration in the lung and exuberant inflammation among the ILD cohort, leading to lung injury or PVD [51,52]. As mentioned previously, under ILD with lower levels of ACE2 molecules, virus invasion aggravates ACE2 deficiency, especially with superinfections, such as bacteria [51,52]. Thus, regardless of the duration of HCQ use, if an infection is out of control, the suboptimal levels of ACE2 with a remnant virus may aggravate the ACE/ACE2 imbalance [16,49,52]. Altogether, the virus may aggravate the primary effect or their comorbidities, thus leading to a higher aHR for PVD (see Figure 6, Table 5) [53].

### 4.6. Women for Higher aHR

Women patients in the 40–49 age group have the highest prevalence of ILD-lupus. Meanwhile, the women seem to have a higher frequency of retinopathy, especially in diabetes or young women with the discontinuation of the HCQ [54,55]. Moreover, in the previous report, the women are more susceptible to the development of PAH. Furthermore, women PAH patients display better right ventricle function and increased survival compared to their men counterparts, a phenomenon referred to as the “estrogen paradox” or “estrogen puzzle” of PAH [56]. Thus, the lower HCQ concentration was associated with the flare-up of lupus with PVD. Meanwhile, young women have an adequate life span for PVD. With a combination of these factors, the young women have a relatively much higher rate in parallel with the higher aHR for PVD. There was also another peak of prevalent cases of lupus in men in the very old age group of 70–79. In contrast, the flare-up of lupus, even with discontinuation, seemed to be non-significant in the elderly [57]. Perhaps, these elderly men may die owing to other diseases, such as cancer (6.78%). Thus, for elderly men, their life span is not adequate to obtain these risks [57]. Taken together, elderly men were not associated with a higher aHR (null effect) for PVD in ILD-lupus.

### 4.7. Highlights of This Study

Altogether, HCQ users have a higher aHR for PVD than the HCQ nonusers, especially at the medium, higher durations or for young women in the ILD cohort. Nevertheless, the confounding factors, such as age, sex, and suboptimal HCQ use, such as discontinuation and perhaps virus, may be taken into account (Table 5).

## 5. Strengths

This novel study investigated the risks HCQ poses to ILD patients, particularly those with immunomodulatory diseases in the general population. We also performed comparisons following propensity score matching to avoid basal line bias, as well as in a time-dependent analysis to minimize immortal time bias. Furthermore, we stratified the treatment of duration times into 28–30, 31–120, and >120 days to minimize lag-time bias and replaced lifestyle with associated comorbidities. For example, exercise and lifestyle were substituted with alcohol-related diseases, hypertension, and hyperlipidemia, a diet with gout and colchicine use, air pollution with stroke, occupation with a sleep disorder, mental disorder or economic condition and smoking with ICSs or OSs use or COPD. Finally, the criteria for the diagnosis and follow up of the ILD cohort patients were strictly followed. For example, we followed up patients with severe acute respiratory syndrome (SARS) using IL-6, a chest X-ray or computed tomography [58]. We did not evaluate HCQ levels (which are not part of the usual care at most hospitals) or self-reported adherence. Nevertheless, in adjusting for sex, age, comorbidities, multiple medications, virus infection-related code and different duration (short-, medium-, long-term duration) entry into the sensitivity analysis, we accounted for factors that are themselves strong predictors of adherence and HCQ concentration.

## 6. Limitations

Biochemistry data were unavailable in the NHIRD. However, although ILD was classified as a catastrophic disease, not all drugs for ILD, such as antifibrotic drugs, interferon, anti-interleukin, imuran and NSAIDs drugs were included in the analysis. Nevertheless, biochemistry data, such as IL-6, were collected in the following sessions in the course of ILD and SARS in Taiwan to facilitate the monitoring of drug use in immunodeficiency diseases [59]. The results of the present study are representative of the population in the real world. However, the true prevalence of the virus infection was unavailable in the NHIRD. The impact of HCQ use on PVD in ILDs with virus infection needs to be further researched in the future. In IPF, immunosuppression is not a first-line treatment in non-licensed therapy in CTD-ILD. We did not perform a subgroup analysis for IPF. Finally, no formal criteria for assessing the PF in patients with ILDs exists. This was another limitation of the study.

## 7. Conclusions

The medium- or long-term use of HCQ for young women who received HCQ were associated with a higher aHR for PVD in ILD with virus infection. These findings indicated the interplay of the primary immunologic effect of ILD, comorbidities, women, age and virus in the HCQ users cohort.

## Figures and Tables

**Table 1:** Demographic characteristics and comorbidities in the propensity score-matched cohorts with and without hydroxychloroquine used among patients with interstitial lung disease with virus infection.

	No Matched			Propensity Score-Matched		
	Hydroxychloroquine			Hydroxychloroquine		
	No	Yes		No	Yes	
Variable	N = 95,081	N = 4703	*p*-Value	N = 4703	N = 4703	*p*-Value
**Age, year**			<0.001			0.70
≤49	44,313 (46.6)	2285 (48.6)		2244 (47.7)	2285 (48.6)	
50–64	31,521 (33.2)	1623 (34.5)		1648 (35.0)	1623 (34.5)	
65+	19,247 (20.2)	795 (16.9)		811 (17.2)	795 (16.9)	
Median ± (IQR) ^†^	51.3 (39.9–62.1)	50.4 (39.3–60.5)	<0.001	50.8 (38.8–60.9)	50.5 (39.3–60.5)	0.63
**Sex**			<0.001			0.67
Women	49,319 (51.9)	3720 (79.1)		3737 (79.5)	3720 (79.1)	
Men	45,762 (48.1)	983 (20.9)		966 (20.5)	983 (20.9)	
**Comorbidity**						
COPD	21,753 (22.9)	1254 (26.7)	<0.001	1190 (25.3%)	1195 (25.4%)	0.77
Sleep disorder	46,885 (49.3)	2879 (61.2)	<0.001	2946 (62.6)	2879 (61.2)	0.15
Diabetes and retinopathy	15,622 (16.4)	488 (10.4)	<0.001	474 (10.1)	488 (10.4)	0.63
Hypertension	54,828 (57.7)	2263 (48.1)	<0.001	2336 (49.7)	2263 (48.1)	0.13
Hyperlipidemia	66,739 (70.2)	2219 (47.2)	<0.001	2269 (48.3)	2219 (47.2)	0.30
Alcohol-related illness	5786 (6.09)	188 (4.00)	<0.001	155 (3.30)	188 (4.00)	0.07
Chronic kidney disease	8933 (9.40)	425 (9.04)	0.41	373 (7.93)	425 (9.04)	0.05
CAD	32,275 (33.9)	1539 (32.7)	0.08	1592 (33.9)	1539 (32.7)	0.25
Stroke	8271 (8.70)	320 (6.80)	<0.001	351 (7.46)	320 (6.80)	0.21
Cancer	6890 (7.25)	319 (6.78)	0.23	271 (5.76)	319 (6.78)	0.04
Gout	25,033 (26.3)	1133 (24.1)	0.001	1150 (24.5)	1133 (24.1)	0.68
Mental disorder	52,391 (55.1)	3087 (65.6)	<0.001	3153 (67.0)	3087 (65.6)	0.15
**Medications**						
Colchicine	21,134 (22.2)	1569 (33.4)	<0.001	1571 (33.4)	1569 (33.4)	0.97
Inhaled corticosteroids (ICSs)	22,223 (23.4)	2054 (43.7)	<0.001	4614 (98.1)	4614 (98.1)	0.99
Other medications	87,254 (91.8)	4614 (98.1)	<0.001	2058 (43.8)	2054 (43.7)	0.93

Chi-square test; ^†^: Mann–Whitney test. CAD: coronary artery disease COPD: chronic obstructive pulmonary disease with virus infection. Other medications—aspirin, statins; anticoagulants—warfarin, heparin, clopidogrel; nonsteroid anti-inflammatory drugs (NSAIDs); and immunosuppressants—cyclophosphamide, azathioprine, methotrexate.

**Table 2:** Incidence and HRs of pulmonary vascular disease in the hydroxychloroquine cohorts compared with those in the non-hydroxychloroquine cohorts by Cox proportional hazard models with time-dependent exposure covariates among the interstitial lung disease with virus infection.

	No Matched		Propensity Score Matched	
	Hydroxychloroquine		Hydroxychloroquine	
	No	Yes	No	Yes
	(N = 95,081)	(N = 4703)	(N = 4703)	(N = 4703)
Person-years	707,299	36,075	36,170	36,075
Follow-up time (y), Median ± (IQR)	7.43 (4.19–10.7)	7.75 (4.27–11.2)	7.73 (4.30–11.2)	7.75 (4.27–11.2)
Pulmonary vascular diseases				
Event	542	59	27	59
Rate ^#^	0.76	1.63	0.74	1.63
Crude HR (95% CI)	1 (Reference)	2.14 (1.64, 2.80) ***	1 (Reference)	2.19 (1.39, 3.46) ***
Adjusted HR ^†^ (95% CI)	1 (Reference)	2.14 (1.63, 2.82) ***	1 (Reference)	2.24 (1.42, 3.54) ***

Rate ^#^, incidence rate, per 1000 person-years; Crude HR, relative; Adjusted HR ^†^: multivariable analysis including age, sex, comorbidities and medications; *** *p* < 0.001.

**Table 3:** Incidence and hazards ratio of pulmonary vascular disease measured by age, sex, comorbidity and medications in the hydroxychloroquine cohorts compared with those in the non-hydroxychloroquine cohorts by Cox proportional hazard models with time-dependent exposure covariates among the interstitial lung disease with virus infection.

	No Matched					Propensity Score Matched				
	Hydroxychloroquine					Hydroxychloroquine				
	No		Yes			No		Yes		
	(N = 95,081)		(N = 4703)			(N = 4703)		(N = 4703)		
Variables	Event	Rate ^#^	Event	Rate ^#^	Adjusted HR ^†^ (95% CI)	Event	Rate ^#^	Event	Rate ^#^	Adjusted HR ^†^ (95% CI)
Pulmonary vascular diseases										
**Age, years**										
**≤49**	86	0.24	18	0.96	3.72 (2.17, 6.37) ***	1	0.05	18	0.96	16.9 (2.26,127.3) ***
**50–64**	168	0.74	14	1.14	1.33 (0.76, 2.32)	9	0.72	14	1.14	1.53.33 (0.66, 3.56)
**65+**	288	2.28	27	5.37	2.09 (1.40, 3.12) ***	17	3.17	27	5.37	1.61 (0.87, 2.97)
**Sex**										
Women	297	0.79	47	1.63	2.34 (1.71, 3.22) ***	19	0.65	47	1.63	2.54 (1.49, 4.35) ***
Men	245	0.74	12	1.65	1.61 (0.90, 2.90)	8	1.12	12	1.65	1.33 (0.53, 3.30)

Rate ^#^, incidence rate, per 1000 person-years; Adjusted HR ^†^: multivariable analysis including age, sex, comorbidities and medications; *** *p* < 0.001.

**Table 4:** Incidence and adjusted hazard ratio pulmonary vascular disease stratified by duration for incident event in patients with interstitial lung disease with virus infection.

	N	Event	Person-Year	Rate	Adjusted HR (95% CI) ^†^
Pulmonary vascular diseases					
Non-hydroxychloroquine Use	95,081	542	709,948	0.76	1.00
Duration of Hydroxychloroquine Use ^#^					
28–30 days ^※^ (short-term)	2425	20	20,837	0.96	1.27 (0.81, 1.99)
31–120 days ^※※^ (medium-term)	1022	18	7528	2.39	3.00 (1.87, 4.81) ***
>120 days ^※※※^ (long-term)	1256	21	7776	2.70	3.83 (2.46, 5.97) ***

^#^ The cumulative use days are partitioned into 3 segments by median and third quartile. ^†^ Adjusted HR: multivariable analysis including age, sex, comorbidities and medications; *** *p* < 0.001. **Discontinuation** was defined as patients who discontinued the index treatment and had a prescription gap of 56 consecutive days or more (grace period) after the date of the previous prescription plus drug supply days. **^※^** frequency of the discontinuation (n = 0) in short-term use **^※※^** frequency of the discontinuation (n = 1) in medium-term use **^※※※^** frequency of the discontinuation (≥1) in long-term use.

**Table 5:** Summary of speculations and findings.

	Without Stratification	aHR	With Stratification	aHR ^♥^ Short	aHR Medium	aHR ^♥♥^ Long
**1. Underdiagnosis**		-~0		-	0	0
examination ^※^		-~+		-	0	+
Admission ^¥^		-~+		-	0	+
**2. Life span**						
Inadequate (elderly, fetal)		-		-		
Adequate (young, not fetal)		+			0~+	+
**3. Infection (VIRUS)**						
Under control		0		0	0	0
Out of control		+		+	+	+
**4. Comobordities**		++		0~+	+	++
**5. HCQ**						
Achieve effect ^#^		0~-		0	0~-	-
Optimal high		0~-		0	0~-	-
Fluctuation ^§^		0~+		0	0~+	+
Low		0~+		0	0~+	+
**6.Adverse reaction ^&^**		0~+		0	0~+	+
**Total effect**		+		0	+	++

**Total effects: 1 + 2 + 3 + 4 + 5 + 6. are associated with “-” decreased aHR, “+” increased aHR, “0” null effect on aHR.** ^♥♥^ Notably, long-term use with higher frequency of the examination in parallel with higher frequency of adverse reaction or discontinuation (+aHR) and if infection is out of control (+aHR). ^♥^ In contrast, in short-term use, the HCQ users died of other diseases, such as CAD or fetal virus infection before PVD (-aHR). ^※^ Long-term use increased medical visits (chest x-ray, computed tomography, arterial blood gas, D-dimer), increased the frequency of detecting the risks and (+aHR). Rarely adverse reaction was found within <120 days, patients rarely receiving examination, such as for arterial blood gas, D-dimer within <120 days, especially <30 days (-aHR). ^¥^ Admission (≤1) lower frequency of admission for diagnosis (-aHR), admission (>1) (0 aHR), admission (>2) (+aHR). ^#^ Short-term use without optimal therapeutic concentration has (0 aHR), long-term use with optimal therapeutic concentration (-aHR). ^§^ Fluctuation, no discontinuation in short-term (n = 0), rare discontinuation in medium-term (n = 1) have (0 aHR), discontinuation (n > 1) in long-term have (+aHR). ^&^ Long-term use may have adverse reactions (aHR 0~+).

## Data Availability

The dataset used in this study is held by the Taiwan Ministry of Health and Welfare (MOHW). The Ministry of Health and Welfare must approve our application to access this data. Any researcher interested in accessing this dataset can submit an application form to the Ministry of Health and Welfare, requesting access (MOHW, Email: stcarolwu@mohw.gov.tw). Taiwan Ministry of Health and Welfare address: No.488, Sec. 6, Zhongxiao E. Rd., Nangang Dist., Taipei City 115, Taiwan (R.O.C.). Phone: +886-2-8590-6848. All relevant data are within the paper.

## Abbreviations

HCQ: hydroxychloroquine; PVD: pulmonary vascular disease; ILD: interstitial lung disease; aHRs: adjusted hazard ratios; CI: confidence interval; ICS: inhaler corticosteroid; OS: oral steroid; CXR: chest X-rays; SARS: severe acute respiratory syndrome; IL-6: interleukin-6; NHIRD: National Health Insurance Registration Database; ICD-9-CM: International Classification of Diseases, Ninth Revision, Clinical Modification.

## Appendix A

**Table A1:** Adverse reaction of Hydroxychloroquine.

Ophthalmological Reactions	Retinopathy (progressive and irreversible changes) is usually associated with long-term use (>5 years), although it can occur earlier.
Cardiomyopathy and QT Prolongation	Cardiac arrest, cardiac failure, cardiomyopathy, endocarditis and myocarditisDetected early, cardiomyopathy may be reversible
Haematological Reactions	Bone marrow depression (aplastic anemia, leucopenia, thrombocytopenia, agranulocytosis) and porphyria exacerbation.
Neurological and Neuromuscular Reactions	Peripheral neuropathyNeuromyopathy (proximal muscle weakness, diminished tendon reflexes, may be reversible on discontinuation of treatment)

**Table A2:** Full name of ICD-9CM with interstitial lung disease with virus infection and comorbidities.

OUTCOMES	
415	acute pulmonary heart disease
415.1	pulmonary embolism and infarction
416	chronic pulmonary heart disease
416.0	pulmonary artery hypertension
416.2	chronic pulmonary embolism
417	other diseases of pulmonary circulation
Autoimmune disease—ILD	
135	sarcoidosis
237.7	neurofibromatosis
272.7	lipidoses
277.3	amyloidosis
277.8	other specified disorders of metabolism (including eosinophilic granuloma)
446.21	Goodpasture’s syndrome
446.4	Wegener’s granulomatosis
Environment—ILD	
495	extrinsic allergic alveolitis (EAA, HP)
500	coal workers’ pneumoconiosis
501	asbestosis
502	pneumoconiosis due to other silica or silicates
503	pneumoconiosis due to other inorganic dust
504	pneumonopathy due to inhalation of other dust
505	pneumoconiosis (unspecified)
506.4	chronic respiratory conditions due to chemicals, gases, fumes, and vapors
508.1	chronic and other pulmonary manifestations due to radiation
508.8	respiratory conditions due to other specified external agents
ILD-VIRUS	
515	postinflammatory pulmonary fibrosis
516	other alveolar and parietoalveolar pneumonopathy, which includes
516.30	idiopathic interstitial pneumonia not otherwise specified
516.31	idiopathic pulmonary fibrosis (IPF)
516.32	idiopathic nonspecific interstitial pneumonitis(iNSIP)
516.33	acute interstitial pneumonitis
516.34	respiratory bronchiolitis interstitial lung disease
516.35	idiopathic lymphoid interstitial pneumonia
516.36	cryptogenic organizing pneumonia
516.37	desquamative interstitial pneumonia
Connective tissue disease (CTD)—ILD	
517.2	lung involvement in systemic sclerosis
517.8	lung involvement in other diseases classified elsewhere;
518.3	pulmonary eosinophilia
555	Crohn’s disease -
710	diffuse diseases of connective tissue
710.0	lupus
710.1	systemic sclerosis
710.2	Sjögren’s disease
710.3	dermatomyositis
710.4	polymyositis
714.81	rheumatoid lung
720	ankylosing spondylitis and other inflammatory spondylopathies
759.5	tuberous sclerosis
Comorbidities	
cataract	
362	retinal disorders
491,492,496 COPD	chronic obstructive pulmonary disease, virus?
307.4, 780.5	sleep disorder, virus?
205	diabetes
401–405	hypertension
272	hyperlipidemia
291, 303, 305, 503.81, 571.0–571.3, and 790.3	alcohol-related illness
585	chronic kidney disease
410–414	CAD
430–438	stroke
140–208	cancer
274	gout
290–319	mental disorder
Virus infection coexists with ILD	
042.0	Human immunodeficiency virus [HIV] disease
053.0	herpes virus
070.20 070.22070.30 070.32	hepatitis B
070.41 070.44.070.51 070.54.	Hepatitis C
075.0	Epstein-Barr virus
078.5	cytomegalic infection
079.0	adenovirus infection
079.1	echo virus infection
079.2	rhinovirus infection
079.3	human papillomavirus
079.4	retrovirus in conditions
079.5	respiratory syncytial virus (RSV)
079.81	hantavirus infection
079.82	SARS-associated coronavirus
079.83	parvovirus B19
079.88	other specified chlamydial infection
079.89	unspecified viral infections
480	virus pneumonia
486	pneumonia (including bacteria)
487–488	Influenza
ATC	drug names
H02A	oral steroids
M01A	NSAIDs
C10AA	statins
B01AC06	aspirin
R03BA	inhaled corticosteroids
L01AA01	Cyclophosphamide (CYC)
L04AX01	Azathioprine (AZA)
L04AX03	Methotrexate (MTX)
L04AB06	TNF-α antagonists
L04AD01	cyclosporine
B01AA03	warfarin
B01AC04	clopidogrel
B01AB01	heparin
